# Clinical Features of Emergency Department Patients from Early COVID-19 Pandemic that Predict SARS-CoV-2 Infection: Machine-learning Approach

**DOI:** 10.5811/westjem.2020.12.49370

**Published:** 2021-03-04

**Authors:** Eric H. Chou, Chih-Hung Wang, Yu-Lin Hsieh, Babak Namazi, Jon Wolfshohl, Toral Bhakta, Chu-Lin Tsai, Wan-Ching Lien, Ganesh Sankaranarayanan, Chien-Chang Lee, Tsung-Chien Lu

**Affiliations:** * Baylor Scott & White All Saints Medical Center, Department of Emergency Medicine, Fort Worth, Texas; † National Taiwan University Hospital, Department of Emergency Medicine, Taipei, Taiwan; ‡ National Taiwan University, College of Medicine, Department of Emergency Medicine, Taipei, Taiwan; § Danbury Hospital, Department of Internal Medicine, Danbury, Connecticut; ¶ Baylor Scott & White Research Institute, Dallas, Texas; || Baylor University Medical Center, Center for Evidence Based Simulation, Dallas, Texas; # Texas A&M Health Science Center, Department of Surgery, Dallas, Texas

## Abstract

**Introduction:**

Within a few months coronavirus disease 2019 (COVID-19) evolved into a pandemic causing millions of cases worldwide, but it remains challenging to diagnose the disease in a timely fashion in the emergency department (ED). In this study we aimed to construct machine-learning (ML) models to predict severe acute respiratory syndrome coronavirus-2 (SARS-CoV-2) infection based on the clinical features of patients visiting an ED during the early COVID-19 pandemic.

**Methods:**

We retrospectively collected the data of all patients who received reverse transcriptase polymerase chain reaction (RT-PCR) testing for SARS-CoV-2 at the ED of Baylor Scott & White All Saints Medical Center, Fort Worth, from February 23–May 12, 2020. The variables collected included patient demographics, ED triage data, clinical symptoms, and past medical history. The primary outcome was the confirmed diagnosis of COVID-19 (or SARS-CoV-2 infection) by a positive RT-PCR test result for SARS-CoV-2, and was used as the label for ML tasks. We used univariate analyses for feature selection, and variables with P<0.1 were selected for model construction. Samples were split into training and testing cohorts on a 60:40 ratio chronologically. We tried various ML algorithms to construct the best predictive model, and we evaluated performances with the area under the receiver operating characteristic curve (AUC) in the testing cohort.

**Results:**

A total of 580 ED patients were tested for SARS-CoV-2 during the study periods, and 98 (16.9%) were identified as having the SARS-CoV-2 infection based on the RT-PCR results. Univariate analyses selected 21 features for model construction. We assessed three ML methods for performance: of the three methods, random forest outperformed the others with the best AUC result (0.86), followed by gradient boosting (0.83) and extra trees classifier (0.82).

**Conclusion:**

This study shows that it is feasible to use ML models as an initial screening tool for identifying patients with SARS-CoV-2 infection. Further validation will be necessary to determine how effectively this prediction model can be used prospectively in clinical practice.

## INTRODUCTION

Within a few months, coronavirus disease 2019 (COVID-19) evolved into a major pandemic causing millions of cases worldwide.^1^–^2^ Early detection of severe acute respiratory syndrome coronavirus 2 (SARS-CoV-2), the viral agent that causes COVID-19 disease, is essential for patient isolation, treatment, and containment of the virus to prevent its further community spread. In the absence of reliable screening tools, emergency physicians have to rely on patients’ clinical symptoms, travel, and contact histories to determine whether they are suitable candidates to have the molecular diagnostic tests for SARS-CoV-2. At present, reverse transcriptase polymerase chain reaction (RT-PCR) remains the gold standard to detect the presence of SARS-CoV-2.^3^–^4^ However, it takes between 4–8 hours to obtain the test result, and may take up to two days or even a week because of the time spent for sample transport to the lab.^5^–^7^ Additionally, the process of sample collection, transport, and communication of results can be labor intensive and subject to human error.

People with COVID-19 may have a wide range of clinical symptoms ranging from mild to severe illness. Since the symptoms of COVID-19 are similar to other viral respiratory illnesses, an emergency approach to COVID-19 should focus on identifying and isolating patients at risk for infection.^8^ Several published reports have described using patients’ symptoms to develop a prediction model for identifying SARS-CoV-2 infections.^9^–^11^ Nonetheless, almost all of the constructed models rely on the combination of symptoms and laboratory/radiological exams to develop the model, which may increase the risk of virus exposure to healthcare providers.

With the advancement of information technology, researchers and clinicians have also sought to develop artificial intelligence (AI)- or machine learning (ML)-based diagnostic tools for detecting COVID-19, but they are either focused on the patients visiting the local clinic or individuals in the community.^12^–^13^ For COVID-19 prediction in emergency department (ED) patients using an ML approach, most of the researchers constructed their models by using a combination of symptoms, laboratory data, and image findings.^14^–^15^ In this study, we attempted to investigate the potential of constructing ML models to predict SARS-CoV-2 infection based on clinical features alone from patients visiting a single ED during the early COVID-19 pandemic. The feasibility for clinical application is also discussed.

## METHODS

### Study Design and Setting

We conducted a retrospective cohort study with data retrieved from the electronic health record (EHR) over the study period (from February 23, 2020, the first case of RT-PCR testing for SARS-CoV-2 in our ED, to May 12, 2020) at the ED of Baylor Scott & White All Saints Medical Center, a 574-bed university-affiliated tertiary care teaching hospital with approximately 50,000 ED visits annually. This study followed the Standards for Reporting of Diagnostic Accuracy statement: explanation and elaboration.^16^

**Population Health Research Capsule**

What do we already know about this issue?

*Diagnosing coronavirus disease 2019 (COVID-19) in a timely fashion was challenging in the emergency department during the early pandemic*.

What was the research question?

Is machine learning (ML) a feasible method to predict COVID-19 based only on clinical features from patients visiting the ED?

What was the major finding of the study?

*We successfully constructed ML models to predict SARS-CoV-2 infection based on the clinical features alone for ED patients*.

How does this improve population health?

*ML has the potential to serve as a screening tool to identify ED patients at risk of SARS-CoV-2 infection*.

### Selection of Participants and Methods of Measurement

In this study we identified all patients with suspected COVID-19 who were tested for SARS-CoV-2 using RT-PCR technique. Samples for RT-PCR tests were taken from the upper (nasopharyngeal or oropharyngeal swabs) respiratory tract, and assayed by using the cobas SARS-CoV-2 Test (Roche Molecular Systems, Inc., Pleasanton, CA). We included only patients attending the ED. Patients without ED triage data were excluded from the analysis. The decision to perform the RT-PCR test for SARS-CoV-2 was left to the discretion of the emergency physicians or physician assistants who cared for the patient. There was no intervention in this study. This study was institutional review board-approved by the Baylor Scott & White Research Institute.

Patient demographics, including age, gender, race, insurance status, weight, height, body mass index (BMI), smoking, and past medical histories (PMH), were obtained from the EHR. We also extracted data on oxygen supplied (yes or no) at ED triage and other ED triage data, including the five-level triage acuity, emergency medical services (EMS) transport (yes or no), Glasgow Coma Scale (GCS) score, body temperature, pulse rate, respiratory rate, oxygen saturation (SpO_2_), duration of symptoms before presentation, travel (to areas with ongoing community transmission of SARS-CoV-2), and contact (in close contact with a confirmed or probable case of COVID-19) histories. Clinical symptoms were manually retrieved from the narrative patient records (including chief complaints and history of present illness) and review of systems recorded by a template with structural format during patient encounters. A total of 36 different clinical symptoms were included for analyses in this study. The primary outcome was the confirmed diagnosis of COVID-19 (or SARS-CoV-2 infection), defined as a positive RT-PCR test result for SARS-CoV-2. The positivity rate for COVID-19 was calculated as the number of positive results divided by the number of tests ordered in patients presenting to the ED.

### Primary Data Analysis

After data collection and preparation, we included the features (variables) as listed above. Missing values in variables were retrieved by a research assistant from the patient’s EHR, or replaced with imputed values if no substantial missing rate (<10%) in that specific variable. The binary outcome of SARS-CoV-2 infection was designated as the classification label. We split the dataset into the training and testing cohorts by time of presentation at a ratio of 60:40 to simulate a prospective validation of the derived model. Data were entered and processed with Microsoft Excel 2010 (Microsoft Corporation, Redmond, WA) and then analyzed with IBM SPSS Statistics for Windows version 24.0 (IBM Corporation, Armonk, NY). We reported results as mean with standard deviation for continuous variables, percentages for categorical variables, and median with interquartile range for time variables.

We used univariate analyses (outcome differences between groups evaluated with Student’s t-test, chi-squared test, Fisher’s exact test, or Mann–Whitney U test depending on the distribution) as the feature selection strategy, and we selected variables with *P*<0.1 in the training cohort as the input features for constructing the ML models. Supervised ML algorithms using random forest, gradient boosting, and extra trees classifier were employed to construct the prediction models. Models were trained in the training cohort and performances were evaluated in terms of area under the receiver operating characteristic curve (AUC) on the testing cohort. We also reported the classification performances on the testing cohort using accuracy, F1-score, precision (or positive predictive value [PPV]), recall (sensitivity), specificity, negative predictive value (NPV), and area under the precision-recall curve, also known as average precision (AP), for each model. All ML analyses were performed using Python 3.8 programming language (Python Software Foundation, Wilmington, DE) with package scikit-learn 0.23.1 installed.^17^

## RESULTS

We retrieved a total of 598 cases from the EHR system during the targeted study period. After excluding those non-ED patients or patients without ED triage data, we identified 580 cases receiving the RT-PCR testing for SARS-CoV-2. Of them, 98 were confirmed to have the SARS-CoV-2 infection based on the RT-PCR results. The positivity rate of COVID-19 in this cohort was 16.9%. Missing data ranged from a low of 0% for most of the variables to a high of 7.6% for BMI. There were 36 cases (6.2%) with travel history and 110 (19.0%) with contact history. Of the 36 included symptoms, shortness of breath was the most common symptom (334, 57.6%), followed by fever (266, 45.9%) and cough (362, 26.4%). The training cohort consisted of 348 cases presented to our ED from February 23–April 14, 2020, while the testing cohort consisted of 232 cases from April 14–May 12, 2020. The characteristics of the study population are shown in Supplementary Table 1.

The characteristics and univariate analyses of variables (features) between patients with or without COVID-19 are summarized in Supplementary Table 2, for the training and testing cohorts, respectively. We selected a total of 21 features by setting the *P*-value of less than 0.10 from the training cohort, including four demographics (race, weight, BMI, smoking history); six triage data (EMS transport, temperature, respiratory rate, oxygen saturation, travel history, contact history); seven symptoms (altered mental status, fever, myalgia, sore throat, hypogeusia/ageusia, cough, diarrhea); and four PMH (comorbidities if any, chronic obstructive pulmonary disease, cerebrovascular accident, depression).

**Figure 1:**
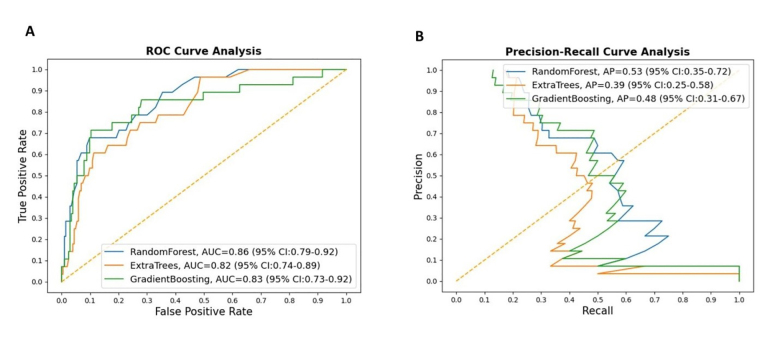
Results of the machine-learning models on the test cohort. (A), Receiver operating characteristic (ROC) curves and the comparison of area under the curve (AUC); (B), Precision-recall curve and the comparison of average precision (AP) for three different machine-learning models.

**Figure 2:**
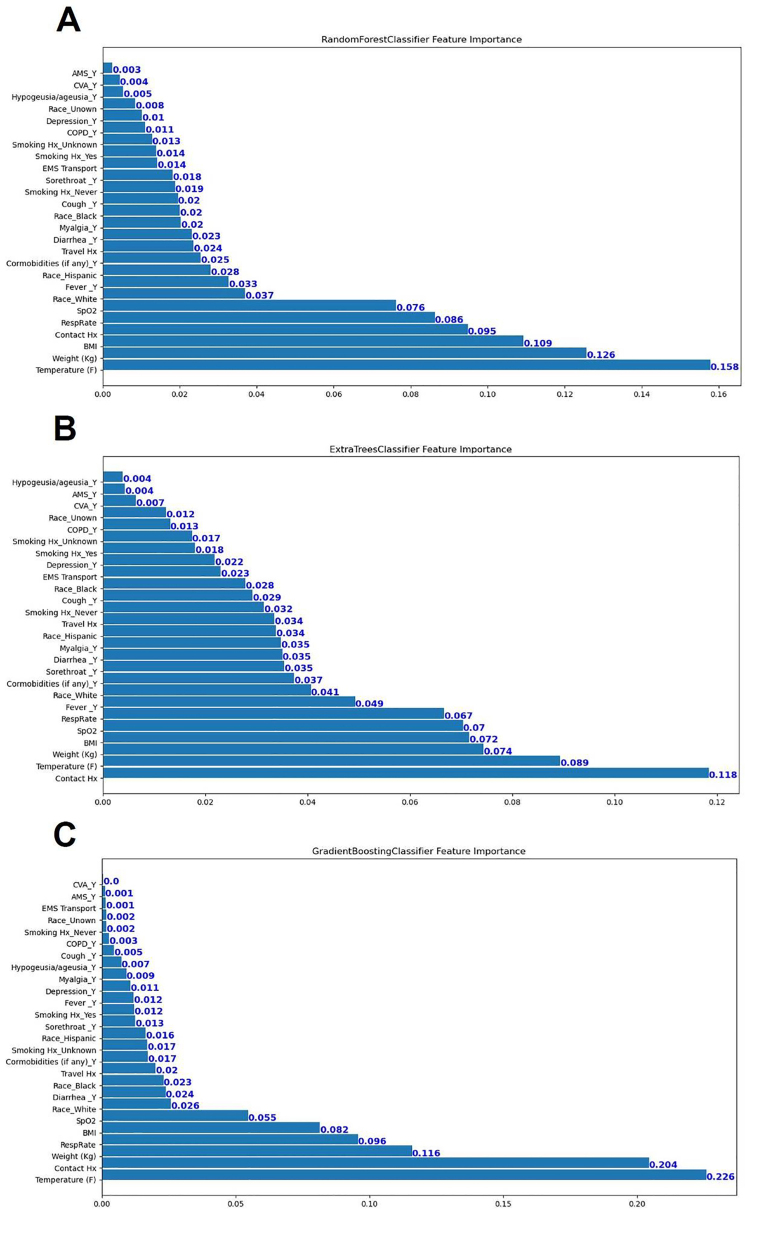
Feature importance for three different machine-learning models: (A), random forest; (B), gradient boosting; and (C), extra trees classifier.

Classification results on the testing cohort for the three different ML models are presented in Table 1 and Figure 1. The top classifier in terms of AUC was random forest (0.86), followed by gradient boosting (0.83), and extra trees classifier (0.82). While adjusting the tradeoff between precision and recall for different thresholds to calculate the AP, random forest (0.53) performed better than gradient boosting (0.48) and extra trees classifier (0.39). However, differences between each model in terms of AUC and AP were not significant. When considering the other performance measures (except recall, or sensitivity), random forest also outperformed the other two ML models in terms of accuracy, F1-score, precision (PPV), specificity, and NPV. Figure 2 shows the feature importance for three different ML models and their feature scores. Of them, all of the three ML models selected temperature, weight, BMI, contact history, respiratory rate, and SpO_2_ as their most important features for the construction of the prediction models.

## DISCUSSION

In this study, we applied ML techniques to predict SARS-CoV-2 infection from patients who visited the ED during the early months of the COVID-19 pandemic. By using 21 clinical features available at ED encounters, we successfully built ML models capable of classifying the risk of SARS-CoV-2 infection. Instead of using base models like decision tree learning, all of the models we used in this study were ensemble methods, which are ML methods that construct a set of predictive models and combine their outputs into a single prediction to achieve better predictive performance.^18^ Our use of the advanced ML algorithms allowed for achieving good predictive performances and also identifying more clinical variables related to the diagnosis of COVID-19. Using AUC as the performance indicator, random forest outperformed gradient boosting and extra trees classifier when applied to the testing cohort. The leading features recognized by these ML models – temperature, weight, BMI, contact history, respiratory rate, and SpO_2_ – are discussed below.

### Comparison with Previous Studies

The spectrum of symptoms caused by COVID-19 ranges from mild to critical; most patients’ symptoms are not severe and they may even be asymptomatic,^19^–^22^ which makes it difficult for clinicians to differentiate COVID-19 from other common respiratory diseases. Novel technology may facilitate timely identification of possible patients to deploy appropriate interventions. The potential employment of ML in clinical practice for detecting and predicting the coronavirus (CoV) family has been previously discussed.^23^ In a review published in May 2020 Albahri et al surveyed state-of-the-art techniques for CoV prediction algorithms based on data mining and ML assessment; they found a total of eight articles published between 2016–2019. Of those articles, seven focused on the prediction or identification of Middle East respiratory syndrome (MERS)-CoV and one focused on extracting difference and similarity between SARS-CoV and MERS-CoV.^24^ The most common algorithms and methods used in the literature review were decision tree (5), naïve Bayes (4), support vector machine (4), and k-nearest neighbor (2). Only one study used the random forest algorithm, one of the ensemble methods used in our study. 25

In a multicenter study conducted in China, Mei et al developed AI algorithms to combine findings on chest computed tomography (CT) with clinical symptoms, contact history, and laboratory results to diagnose patients with COVID-19.^5^ In their cohort, the average age was 40.7 years with 46.3% (419/905) of the patients testing positive for COVID-19. Patient’s age, exposure to SARS-CoV-2, fever, cough, cough with sputum, and white blood cell counts were significant clinical features associated with COVID-19. In the joint model combining both clinical data and CT imaging, the AUC achieved 0.92 with 84.3% sensitivity and 82.8% specificity. The convolutional neural network (CNN) model employing only CT imaging data achieved 0.86 AUC with 83.6% sensitivity and 75.9% specificity while the multilayer perceptron (MLP) model incorporating clinical data alone achieved 0.80 AUC with 80.6 % sensitivity and 68.3% specificity.

In comparison with either CNN or MLP models in the Mei et al study, our random forest model achieved 0.86 AUC with clinical features alone. This improved AUC may be caused by the increased number of clinical features incorporated in our model as compared to the model developed by Mei et al.^5^ Despite the fact that their joint model outperformed our random forest model, the employment of CT findings could raise additional concerns. First, the process of CT scanning may be complicated by the infection control protocol, thereby lengthening the time consumed by the radiological procedure.^26^–^27^ Second, whether the convalescent patients could be immune to recurrent infections by SARS-CoV-2 is still debated, but recurrent infections have been reported^28^; therefore, repeat CT scanning with high radiation dose may be both costly and harmful. In contrast, our models incorporated only those clinical variables available at the time of initial ED encounter; therefore, potential COVID-19 patients could be proactively identified and introduced to isolation areas before lab work, imaging or physician evaluation, thus minimizing the risk of person-to-person transmission of SARS-CoV-2 while these patients stayed in the waiting zone.

### Interpretation of Current Study

In a study conducted by Peyrony et al that included a cohort of 391 patients with 57.5% infected with COVID-19, the most commonly reported symptoms were fever, cough, dyspnea, and myalgia.^10^ However, among the collected symptoms and signs, only four of them (myalgia, anosmia, temperature ≥38°C and SpO_2_ <95%) achieved more than 80% specificity. Similarly, features selected to construct the ML models in our studies – temperature, BMI, weight, contact history, oxygen saturation, and respiratory rate – were consistently ranked as the top six important variables in predicting COVID-19. It has been reported that people with elevated BMI or body weight may sustain a more serious SARS-CoV-2 infection.^29^–^31^ Therefore, COVID-19 patients with elevated BMI may have higher chances to receive RT-PCR exam due to severe symptoms or signs, compared with those with lower BMI, resulting in selection bias. Interestingly, in the Peyrony et al study, anosmia was reported in 13.8 % of COVID-19 patients and was identified to be the most specific symptom of SARS-CoV-2 infection (specificity: 98%). In contrast, our cohort reported anosmia in only 3% of COVID-19 patients. Since anosmia and dysgeusia were initially noted among COVID-19 patients in April,^32^ these symptoms may be under-reported in a retrospective study, resulting in reporting bias.

Our study included 580 patients receiving RT-PCR testing for SARS-CoV-2 during late February and early May 2020, among whom, 98 (16.9%) patients tested positive. The training and testing cohorts were divided chronologically to simulate a prospective study; ie, the performance of the ML models were developed on the basis of the past (training) cohort and evaluated in the future (testing) cohort. The study demonstrated excellent AUC results based on the three ML models we used, with the random forest model achieving the best performance in this analysis (0.86). During this period, the policy for performing RT-PCR testing did not change substantially in our hospital and, therefore, the features between training and testing cohorts were quite similar (Supplementary Table 1). Although the proportions of COVID-19 patients differed significantly between training (20.1%) and testing (12.1%) cohorts, our random forest model still achieved excellent classification performance in the testing cohort.

In addition to AUC, we evaluated the performance of our models by using a series of the available performance measures, including specificity, recall (sensitivity), and precision (or PPV). Precision is a measure of how often the predictions for the positive class are actually true, and the goal of a good ML model is to obtain the right balance of precision and recall (Figure 1B). While we obtained very high specificity values for all of the constructed ML models, our results nevertheless showed that the recall values in all of the constructed models were low, implying the models are good for ruling in the disease of interest (COVID-19) rather than ruling it out. Our results showed that the specificity and NPV of the random forest model achieved 0.97and 0.92, respectively, which may be employed to classify the risk of SARS-CoV-2 infection for ED visits if appropriately adjusting the cut-point. Because of the wide spectrum of presented symptoms and signs of COVID-19, it may be difficult for clinicians to determine who should receive RT-PCR testing for SARS-CoV-2. In communities that have limited resources for SARS-CoV-2 testing kits or enough space for isolation, a prediction model with high specificity or NPV may assist clinicians in allocating precious healthcare resources and initiating early intervention for high-risk patients.

### Feasibility for Clinical Application

The COVID-19 pandemic has propagated exponentially because of widespread person-to-person transmission and global transportation.^2^, ^33^ Infection of SARS-CoV-2 is confirmed with RT–PCR exam, but it could take up to a week to get the test results.^7^ Because of the increasing need for testing and isolation, we proposed that an ML algorithm could facilitate triaging the relatively limited healthcare resources in order to halt the progress of the current pandemic. Moreover, since we used only clinical features, which can be obtained immediately at ED triage, suspected patients with COVID-19 could thus be rerouted to isolation areas even before they enter the ED, limiting the potential person-to-person transmission and nosocomial infection.

Getting safe emergency care during the COVID-19 pandemic is of paramount importance both for the patients seeking help when they feel ill and for the healthcare providers in the ED. With appropriate risk stratification, healthcare personnel may thus have fewer risks of exposure to patients with suspected SARS-CoV-2 infection. As the COVID-19 pandemic persists, an ML-assisted prediction algorithm could help slow transmission and more judiciously allocate our finite healthcare resources. To be used as a diagnostic tool for aiding the identification of patients at risk of COVID-19 infection in the near future, technological feasibility assessment has to be conducted before the full implementation of the decision support tool. This assessment should be based on outlining the design of the resource requirements, and understanding the barriers and obstacles related to both science and logistics.^34^

## LIMITATIONS

There are several limitations in this study. First, clinical symptoms were collected retrospectively, which could have been subject to reporting bias. For example, anosmia seemed to be under-reported in our report when compared with other studies.^5^,^10^ Second, this study was conducted in an ED of a single center during the early period of the pandemic with limited sample size. Enrollment was based on patients who were judged to have the need for RT-PCR testing, and not all patients were tested for SARS-CoV-2. Despite the discriminatory performance of the ML models to identify patients with SARS-CoV-2 infection, this approach may introduce selection bias and more data are required to examine the generalizability of the models to other patient populations. Third, in consideration of the dynamic course of the COVID-19 pandemic and the constantly changing policies concerning screening and isolation, the presenting features of COVID-19 patients may also change substantially.

In addition, the fact that we split the dataset into training and testing cohorts by time of presentation may have introduced sampling bias since test availability in the US varied over the study period. Therefore, our proposed model should be proactively updated and adapted to the current patient population. Forth, clinical symptoms were manually retrieved in our study rather than being automatically extracted from the EHR. It is possible that such an approach would be biased in variable selection.

Finally, due to the low positivity rate of COVID-19 in our population, our ML model construction suffered from the problem of imbalanced classification.^35^ It can be a challenging task to report the classification performances with regard to the imbalanced distribution of the dataset. We balanced the data by weighing the samples by the imbalanced ratio, and evaluated the prediction performances of our ML models by using most of the available methods of performance measures, including AUC and AP, to avoid bias or over-interpretation by any of the results. Nevertheless, our study showed fair results in performance measures using recall and kappa, leaving room for future improvement.

## CONCLUSION

We successfully constructed the ML models to predict COVID-19 with excellent discrimination ability based on the clinical features of initial ED encounters. Implementation of this tool may serve as an initial screening tool for identifying patients at risk of SARS-CoV-2 infection. Further validation will be necessary to determine how effectively this prediction model can be used prospectively in clinical practice.

## Supplementary Information



## Figures and Tables

**Table 1:** Comparison between model performances on the testing cohort.

Models	AUC	AP	Accuracy	F1-score	Kappa	Recall (Sensitivity)	Specificity	PPV (Precision)	NPV
Random Forest	0.86	0.53	0.89	0.39	0.27	0.29	0.98	0.58	0.92
Gradient Boosting	0.83	0.48	0.88	0.36	0.34	0.29	0.96	0.50	0.91
Extra Trees Classifier	0.82	0.39	0.86	0.36	0.27	0.32	0.94	0.41	0.91

*AUC*, area under the receiver operating characteristic curve; *AP*, area under the precision recall curve (average precision); *PPV*, positive predictive value; *NPV*, negative predictive value.
