# Supplementary material for: Clinical Features of Emergency Department Patients from Early COVID-19 Pandemic that Predict SARS-CoV-2 Infection: Machine-learning Approach
Source: West J Emerg Med. 2021 Mar 4;22(2):244–51. doi: 10.5811/westjem.2020.12.49370 (PMC7972393; doi:10.5811/westjem.2020.12.49370)
Supplement: Supplementary file 1 [file wjem-22-244-s001.docx]

**Supplementary Table 1.** Characteristics of the study population: patients tested in the emergency department for severe acute respiratory syndrome coronavirus-2.

| **Variables (Features)** | | Total  (n = 580) | Training cohort  (n = 348) | Testing cohort  (n = 232) | *P* value |
| --- | --- | --- | --- | --- | --- |
| **Demographics** | | | | | |
|  | **Age (years), Mean (SD)** | 53.7 (18.9) | 54 (19) | 53 (19) | 0.37 |
|  | **Gender**  Male  Female | 213 (36.7)  367 (63.3) | 125 (35.9)  223 (64.1) | 88 (37.9)  144 (62.1) | 0.62 |
|  | **Race**  Asian  Black  Hispanic  White  Unknown | 10 (1.7)  166 (28.6)  113 (19.5)  272 (46.9)  19 (3.3) | 8 (2.3)  99 (28.4)  62 (17.8)  168 (48.3)  11 (3.2) | 2 (0.9)  67 (28.9)  51 (22.0)  104 (44.8)  8 (3.4) | 0.51 |
|  | **Insurance Status**  Commercial  Medicaid/Medicare  None  Unknown | 308 (53.1)  155 (26.7)  114 (19.7)  3 (0.5) | 182 (52.3)  92 (26.4)  71 (20.4)  3 (0.9) | 126 (54.3)  63 (27.2)  43 (18.5)  0 (0) | 0.50 |
|  | **Weight (Kg), Mean (SD)** | 88.9 26.1) | 88.9 (24.0) | 88.9 (29.1) | 0.99 |
|  | **Height (M), Mean (SD)** | 1.68 (0.10) | 1.68 (0.10) | 1.68 (0.10) | 0.81 |
|  | **BMI, Mean (SD)** | 31.5 (9.0) | 31.6 (8.7) | 31.3 (9.5) | 0.68 |
|  | **Smoking History**  Former  Never  Yes  Unknown | 102 (17.6)  376 (64.8)  85 (14.7)  17 (2.9) | 63 (18.1)  230 (66.1)  44 (12.6)  11 (3.2) | 39 (16.8)  146 (62.9)  41 (17.7)  6 (2.6) | 0.41 |
| **Triage Data** | | | | | |
|  | **EMS transport** | 151 (26) | 90 (25.9) | 61 (26.3) | 0.91 |
|  | **Triage acuity** |  |  |  | 0.76 |
|  | Level 1 | 5 (0.9) | 3 (0.9) | 2 (0.9) |  |
|  | Level 2 | 149 (25.7) | 84 (24.1) | 65 (28.0) |  |
|  | Level 3 | 416 (71.7) | 254 (73.0) | 162 (69.8) |  |
|  | Level 4 | 9 (1.6) | 6 (1.7) | 3 (1.3) |  |
|  | Level 5 | 1 (0.2) | 1 (0.3) | 0 (0) |  |
|  | **Oxygen supplied at triage** | 70 (12.1) | 38 (10.9) | 32 (13.8) | 0.30 |
|  | **Travel history** | 36 (6.2) | 31 (8.9) | 5 (2.2) | 0.001** |
|  | **Contact history** | 110 (19.0) | 66 (19.0) | 44 (19.0) | 1.00 |
|  | **GCS, Mean (SD)** | 14.8 (1.4) | 14.8 (1.2) | 14.8 (1.6) | 0.51 |
|  | **Temperature (F), Mean (SD)** | 99.0 (1.3) | 99.1 (1.3) | 98.9 (1.2) | 0.22 |
|  | **Pulse Rate, Mean (SD)** | 92.8 (20.3) | 93.2 (20.4) | 92.1 (20.2) | 0.54 |
|  | **Respiratory Rate, Mean (SD)** | 18.8 (3.6) | 18.9 (3.5) | 18.7 (3.8) | 0.47 |
|  | **SBP (mm Hg), Mean (SD)** | 138.0 (25.7) | 137.9 (25.4) | 138.0 (26.1) | 0.98 |
|  | **DBP (mm Hg), Mean (SD)** | 80.2 (16.7) | 80.3 (16.8) | 80.0 (16.5) | 0.85 |
|  | **SpO_2_ (%), Mean (SD)** | 97.4(3.4) | 97.1 (3.8) | 97.8 (2.5) | 0.01** |
|  | **Duration (Days) before presentation, Median (IQR)** | 3 (1-7) | 3 (2-7) | 3 (1-7) | 0.25 |
| **Clinical Symptoms** | | | | | |
|  | **AMS** | 30 (5.2) | 15 (4.3) | 15 (6.5) | 0.25 |
|  | **Seizures** | 4 (0.7) | 2 (0.6) | 2 (0.9) | 0.68 |
|  | **Fever** | 266 (45.9) | 174 (50) | 92 (39.7) | 0.01** |
|  | **Chills** | 84 (14.5) | 50 (14.4) | 34 (14.7) | 0.92 |
|  | **Myalgia** | 131 (22.6) | 87 (25) | 44 (19) | 0.09 |
|  | **Arthralgia** | 11 (1.9) | 3 (0.9) | 8 (3.4) | 0.03** |
|  | **Headache** | 116 (20) | 76 (21.8) | 40 (17.2) | 0.18 |
|  | **Facial pain** | 4 (0.7) | 3 (0.9) | 1 (0.4) | 0.54 |
|  | **Red eyes** | 5 (0.9) | 4 (1.1) | 1 (0.4) | 0.36 |
|  | **Otalgia** | 10 (1.7) | 7 (2) | 3 (1.3) | 0.52 |
|  | **Sore throat** | 81 (14.0) | 52 (14.9) | 29 (12.5) | 0.41 |
|  | **Rhinorrhea** | 26 (4.5) | 20 (5.7) | 6 (2.6) | 0.07 |
|  | **Stuffy nose** | 69 (11.9) | 44 (12.6) | 25 (10.8) | 0.50 |
|  | **Sneezing** | 8(1.4) | 6(1.7) | 2 (0.9) | 0.38 |
|  | **Postnasal drip** | 5 (0.9) | 3 (0.9) | 2 (0.9) | 1.00 |
|  | **Hypogeusia/ageusia** | 3 (0.5) | 2 (0.6) | 1 (0.4) | 0.81 |
|  | **hyposmia/anosmia** | 6 (1) | 5 (1.4) | 1 (0.4) | 0.24 |
|  | **Hoarseness** | 1 (0.2) | 0 (0) | 1 (0.4) | 0.22 |
|  | **Dysphagia** | 6 (1) | 3 (0.9) | 3 (1.3) | 0.62 |
|  | **Cough** | 362 (26.4) | 236 (67.8) | 126 (54.3) | 0.001** |
|  | **Sputum** | 47 (8.1) | 36 (10.3) | 11 (4.7) | 0.02* |
|  | **SOB** | 334 (57.6) | 211 (60.6) | 123 (53.0) | 0.07 |
|  | **Malaise** | 110 (19) | 68 (19.5) | 42 (18.1) | 0.67 |
|  | **Diarrhea** | 65 (11.2) | 40 (11.5) | 25 (10.8) | 0.79 |
|  | **Vomiting** | 66 (11.4) | 36(10.3) | 30 (12.9) | 0.34 |
|  | **Nausea** | 115 (19.8) | 69 (19.8) | 46 (19.8) | 1.00 |
|  | **Anorexia** | 26 (4.5) | 14 (4) | 12 (5.2) | 0.51 |
|  | **Abdominal pain** | 62 (10.7) | 37 (10.6) | 25 (10.8) | 0.96 |
|  | **Chest pain** | 120 (20.7) | 78 (22.4) | 42 (18.1) | 0.21 |
|  | **Hemoptysis** | 6 (1) | 5 (1.4) | 1 (0.4) | 0.24 |
|  | **Skin lesion** | 5 (0.9) | 4 (1.1) | 1 (0.4) | 0.36 |
|  | **Skin itch** | 3 (0.5) | 3 (0.9) | 0 (0) | 0.16 |
|  | **Paresthesia** | 3 (0.5) | 2 (0.6) | 1 (0.4) | 0.81 |
|  | **Back pain** | 38 (6.6) | 18 (5.2) | 20 (8.6) | 0.10 |
|  | **Flank pain** | 15 (2.6) | 8 (2.3) | 7 (3.0) | 0.59 |
|  | **Sickle cell crisis** | 1 (0.2) | 1 (0.3) | 0 (0) | 0.41 |
| **Past Medical Histories** | |  |  |  |  |
|  | **Comorbidities (if any)** | 450 (77.6) | 271 (77.9) | 179 (77.2) | 0.84 |
|  | **Comorbidities (more than 1)** | 355 (61.2) | 216 (62.1) | 139 (59.9) | 0.60 |
|  | **COPD** | 66 (11.4) | 42 (12.1) | 24 (10.3) | 0.52 |
|  | **Asthma** | 99 (17.1) | 60 (17.2) | 39 (16.8) | 0.89 |
|  | **Diabetes Mellitus** | 149 (25.7) | 97 (27.9) | 52 (22.4) | 0.14 |
|  | **Hypertension** | 276 (47.6) | 162 (46.6) | 114 (49.1) | 0.54 |
|  | **CAD** | 55 (9.5) | 35 (10.1) | 20 (8.6) | 0.56 |
|  | **CHF** | 52 (9.0) | 29 (8.3) | 23 (9.9) | 0.51 |
|  | **CVA** | 36 (6.2) | 22 (6.3) | 14 (6.0) | 0.89 |
|  | **Hepatitis B** | 0 (0) | 0 (0) | 0 (0) | NA |
|  | **Hepatitis C** | 11 (1.9) | 5 (1.4) | 6 (2.6) | 0.32 |
|  | **Cirrhosis** | 14 (2.4) | 8 (2.3) | 6 (2.6) | 0.83 |
|  | **Cancer** | 74 (12.8) | 49 (14.1) | 25 (10.8) | 0.24 |
|  | **Current Chemotherapy** | 11 (1.9) | 9 (2.6) | 2 (0.9) | 0.14 |
|  | **CKD** | 76 (13.1) | 45 (12.9) | 31 (13.4) | 0.88 |
|  | **ESRD** | 32 (5.5) | 21 (6) | 11.(4.7) | 0.50 |
|  | **History of solid organ transplant** | 18 (3.1) | 10 (2.9) | 8 (3.4) | 0.70 |
|  | **Immunodeficiency** | 5 (0.9) | 2 (0.6) | 3 (1.3) | 0.36 |
|  | **HIV infection** | 4 (0.7) | 1 (0.3) | 3 (1.3) | 0.15 |
|  | **Rheumatologic diseases** | 17 (2.9) | 14 (4) | 3 (1.3) | 0.06 |
|  | **Dementia** | 11 (1.9) | 6 (1.7) | 5 (2.2) | 0.71 |
|  | **Peptic ulcer disease** | 1 (0.2) | 0 (0) | 1 (0.4) | 0.22 |
|  | **Gastroparesis** | 4 (0.7) | 4 (1.1) | 0 (0) | 0.10 |
|  | **Sickle cell Disease** | 1 (0.2) | 1 (0.3) | 0 (0) | 0.41 |
|  | **Migraine** | 17 (2.9) | 12 (3.4) | 5 (2.2) | 0.37 |
|  | **Fibromyalgia** | 4 (0.7) | 3 (0.9) | 1 (0.4) | 0.54 |
|  | **Chronic pain syndrome** | 24 (4.1) | 14 (4.0) | 10 (4.3) | 0.86 |
|  | **Alcohol abuse** | 5 (0.9) | 1 (0.3) | 4 (1.7) | 0.07 |
|  | **Substance abuse** | 24 (4.1) | 11 (3.2) | 13 (5.6) | 0.15 |
|  | **Depression** | 55 (9.5) | 32 (9.2) | 23 (9.9) | 0.77 |
|  | **Psychiatric disease** | 52 (9.0) | 31 (8.9) | 21 (9.1) | 0.95 |
|  | **Pregnancy** | 19 (3.3) | 8 (2.3) | 11 (4.7) | 0.11 |

*SD,* standard deviation*; BMI*, Body mass index; *kg*, kilogram; *EMS*, emergency medical services; *GCS*, Glasgow Coma Scale; *SBP*, systolic blood pressure; *DBP*, diastolic blood pressure; *SpO_2_*, oxygen saturation; *AMS*, altered mental status; *SOB*, shortness of breath; *COPD*, chronic obstructive pulmonary disease; *CAD*, coronary artery disease; *CHF*, congestive heart failure; *CVA*, cerebrovascular accident; *CKD*, chronic kidney disease; *ESRD*, end stage renal disease; *HIV*, human immunodeficiency virus.

Note: ** *P*<0.05

**Supplementary Table 2.** Characteristics and univariate analyses of variables (features) between patients with or without COVID-19 in the training and testing cohorts.

|  | | Training Cohort  (N = 348) | | *P* value | Testing Cohort  (N = 232) | | *P* value |
| --- | --- | --- | --- | --- | --- | --- | --- |
|  |  | COVID-19 (-)  (n = 278) | COVID-19 (+) (n = 70) |  | COVID-19 (-) (n=204) | COVID-19 (+) (n=28) |  |
| **Demographics** | |  |  |  |  |  |  |
|  | **Age (years), Mean (SD)** | 55 (19) | 53 (18) | 0.41 | 54 (19) | 44 (19) | 0.01* |
|  | **Gender**  Male  Female | 97 (34.9)  181 (65.1) | 28 (40)  42 (60) | 0.43 | 76 (37.3)  128 (62.7) | 12 (42.9)  16 (57.1) | 0.57 |
|  | **Race**  Asian  Black  Hispanic  White  Unknown | 7 (2.5)  74 (26.6)  41 (14.7)  150 (54.0)  6 (2.2) | 1 (1.4)  25 (35.7)  21 (30.0)  18 (25.7)  5 (7.1) | 0.0001* | 2 (1)  54 (26.5)  43 (21.1)  100 (49.0)  5 (2.5) | 0 (0)  13 (46.4)  8 (28.6)  4 (14.3)  3 (10.7) | 0.003* |
|  | **Insurance Status**  Commercial  Medicaid/Medicare  None  Unknown | 146 (52.5)  75 (27)  54 (19.4)  3 (1.1) | 36 (51.4)  17 (24.3)  17 (24.3)  0 (0) | 0.67 | 111 (54.4)  60 (29.4)  33 (16.2)  0 (0) | 15 (53.6)  3 (10.7)  10 (35.7)  0 (0) | 0.02* |
|  | **Weight (Kg), Mean (SD)** | 87.0 (23.4) | 96.4 (25.3) | 0.03* | 88.5 (29.5) | 91.4 (26.4) | 0.63 |
|  | **Height (M), Mean (SD)** | 1.68 (0.10) | 1.68 (0.09) | 0.82 | 1.68 (0.10) | 1.67 (0.13) | 0.39 |
|  | **BMI, Mean (SD)** | 31.0 (8.7) | 34.1 (8.4) | 0.009* | 31.1 (9.6) | 32.7 (8.4) | 0.42 |
|  | **Smoking History**  Former  Never  Yes  Unknown | 55 (19.8)  179 (64.4)  39 (14.0)  5 (1.8) | 8 (11.4)  51 (72.9)  5 (7.1)  6 (8.6) | 0.005* | 38 (18.6)  126 (61.8)  37 (18.1)  3 (1.5) | 1 (3.6)  20 (71.4)  4 (14.3)  3 (10.7) | 0.007* |
| **Triage Data** | |  |  |  |  |  |  |
|  | **EMS transport** | 78 (28.1) | 12 (17.1) | 0.06* | 54 (26.5) | 7 (25.0) | 0.87 |
|  | **Triage acuity** |  |  | 0.19 |  |  | 0.82 |
|  | Level 1 | 1 (0.4) | 2 (2.9) |  | 2 (1) | 0 (0) |  |
|  | Level 2 | 71 (25.5) | 13 (18.6) |  | 58 (28.4) | 7 (25) |  |
|  | Level 3 | 201 (72.3) | 53 (75.7) |  | 141 (69.1) | 21 (75) |  |
|  | Level 4 | 4 (1.4) | 2 (2.9) |  | 3 (1.5) | 0 (0) |  |
|  | Level 5 | 1 (0.4) | 0 (00) |  | 0 (0) | 0 (00) |  |
|  | **Oxygen supplied at triage** | 34 (12.2) | 4 (5.7) | 0.12 | 25 (12.3) | 7 (25.0) | 0.07* |
|  | **Travel history** | 19 (6.8) | 12 (17.1) | 0.007* | 4 (2) | 1 (3.6) | 0.58 |
|  | **Contact history** | 30 (10.8) | 36 (51.4) | <0.0001* | 29 (14.2) | 15 (53.6) | <0.0001* |
|  | **GCS, Mean (SD)** | 14.9 (1.1) | 14.7 (1.7) | 0.38 | 14.8 (1.5) | 14.6 (2.3) | 0.51 |
|  | **Temperature (F), Mean (SD)** | 98.9 (1.2) | 99.8 (1.3) | <0.0001* | 98.9 (1.2) | 99.6 (1.1) | 0.005* |
|  | **Pulse Rate, Mean (SD)** | 92.7 (20.7) | 95.0 (18.9) | 0.41 | 92.1 (20.8) | 92.1 (16.0) | 0.99 |
|  | **Respiratory Rate, Mean (SD)** | 18.8 (3.2) | 19.6 (4.5) | 0.074* | 18.6 (3.7) | 19.2 (4.3) | 0.47 |
|  | **SBP (mmHg), Mean (SD)** | 139.0 (25.8) | 133.8 (23.0) | 0.13 | 138.9 (26.7) | 131.0 (20.4) | 0.13 |
|  | **DBP (mmHg), Mean (SD)** | 80.3 (17.2) | 80.3 (15.1) | 0.99 | 80.2 (17.2) | 79.2 (9.9) | 0.77 |
|  | **SpO_2_ (%),Mean (SD)** | 97.4 (3.5) | 96 (4.7) | 0.008* | 97.9 (2.3) | 96.9 (3.2) | 0.042* |
|  | **Duration (days) before presentation, Median (IQR)** | 3 (1-7) | 5 (2-7) | 0.10 | 3 (1-7) | 3 (2-7) | 0.39 |
| **Clinical Symptoms** | |  |  |  |  |  |  |
|  | **AMS** | 0 (0) | 15 (5.4) | 0.047* | 14 (6.9) | 1 (3.6) | 0.51 |
|  | **Seizures** | 2 (0.7) | 0 (0) | 0.48 | 2 (1) | 0 (0) | 0.60 |
|  | **Fever** | 123 (44.2) | 51 (72.9) | <0.0001* | 74 (36.3) | 18 (64.3) | 0.005* |
|  | **Chills** | 43 (15.5) | 7 (10) | 0.24 | 29 (14.2) | 5 (17.9) | 0.61 |
|  | **Myalgia** | 63 (22.7) | 24 (34.3) | 0.045* | 35 (17.2) | 9 (32.1) | 0.06* |
|  | **Arthralgia** | 3 (1.1) | 0 (0) | 0.38 | 8 (3.9) | 0 (0) | 0.29 |
|  | **Headache** | 59 (21.2) | 17 (24.3) | 0.58 | 35 (17.2) | 5 (17.9) | 0.93 |
|  | **Facial pain** | 3 (1.1) | 0 (0) | 0.38 | 1 (0.5) | 0 (0) | 0.71 |
|  | **Red eyes** | 4 (1.4) | 0 (0) | 0.31 | 1 (0.5) | 0 (0) | 0.71 |
|  | **Otalgia** | 7 (2.5) | 0 (0) | 0.18 | 1 (0.5) | 2 (7.1) | 0.004* |
|  | **Sore throat** | 47 (16.9) | 5 (7.1) | 0.04* | 23 (11.3) | 6 (21.4) | 0.13 |
|  | **Rhinorrhea** | 15 (5.4) | 5 (7.1) | 0.58 | 4 (2) | 2 (7.1) | 0.11 |
|  | **Stuffy nose** | 35 (12.6) | 9 (12.9) | 0.95 | 20 (9.8) | 5 (17.9) | 0.20 |
|  | **Sneezing** | 5 (1.8) | 1(1.4) | 0.83 | 2 (1) | 0 (0) | 0.60 |
|  | **Postnasal drip** | 3 (1.1) | 0 (0) | 0.38 | 1 (0.5) | 1 (3.6) | 0.10 |
|  | **Hypogeusia/ageusia** | 0 (0) | 2 (2.9) | 0.005* | 0 (0) | 1 (3.6) | 0.007* |
|  | **hyposmia/anosmia** | 3 (1.1) | 2 (2.9) | 0.26 | 0 (0) | 1 (3.6) | 0.007* |
|  | **Hoarseness** | 0 (0) | 0 (0) | NA | 1 (0.5) | 0 (0) | 0.71 |
|  | **Dysphagia** | 3 (1.1) | 0 (0) | 0.38 | 3 (1.5) | 0 (0) | 0.52 |
|  | **Cough** | 179 (64.4) | 57 (81.4) | 0.007* | 106 (52.0) | 20 (71.4) | 0.053* |
|  | **Sputum** | 25 (9.0) | 11 (15.7) | 0.10 | 10 (4.9) | 1 (3.6) | 0.76 |
|  | **SOB** | 170 (61.2) | 41 (58.6) | 0.69 | 107 (52.5) | 16 (57.1) | 0.64 |
|  | **Malaise** | 55 (19.8) | 13 (18.6) | 0.82 | 35 (17.2) | 7 (25) | 0.31 |
|  | **Diarrhea** | 26 (9.4) | 14 (20) | 0.01* | 22 (10.8) | 3 (10.7) | 0.99 |
|  | **Vomiting** | 28 (10.1) | 8 (11.4) | 0.74 | 29 (14.2) | 1 (3.6) | 0.12 |
|  | **Nausea** | 51 (18.3) | 18 (25.7) | 0.17 | 41 (20.1) | 5 (17.9) | 0.78 |
|  | **Anorexia** | 10 (3.6) | 4 (5.7) | 0.42 | 11 (5.4) | 1 (3.6) | 0.68 |
|  | **Abdominal pain** | 31 (11.2) | 6 (8.6) | 0.53 | 23 (11.3) | 2 (7.1) | 0.53 |
|  | **Chest pain** | 65 (23.4) | 13 (18.6) | 0.39 | 41 (20.1) | 1 (3.6) | 0.03* |
|  | **Hemoptysis** | 3 (1.1) | 2 (2.9) | 0.26 | 1 (0.5) | 0 (0) | 0.71 |
|  | **Skin lesion** | 4 (1.4) | 0 (0) | 0.31 | 1(0.5) | 0 (0) | 0.70 |
|  | **Skin itch** | 3 (1.1) | 0 (0) | 0.38 | 0 (0) | 0 (0) | NA |
|  | **Paresthesia** | 1 (0.4) | 1 (1.4) | 0.29 | 1 (0.5) | 0 (0) | 0.71 |
|  | **Back pain** | 14 (5.0) | 4 (5.7) | 0.82 | 19 (9.3) | 1 (3.6) | 0.31 |
|  | **Flank pain** | 6 (2.2) | 2 (2.9) | 0.59 | 6 (2.9) | 1 (3.6) | 0.86 |
|  | **Sickle cell crisis** | 0 (0) | 1 (1.4) | 0.20 | 0 (0) | 0 (0 | NA |
| **Past Medical Histories** | |  |  |  |  |  |  |
|  | **Comorbidities**  **(if any)** | 224 (80.6) | 47 (67.1) | 0.02* | 165 (80.9) | 14 (50.0) | 0.0003* |
|  | **Comorbidities**  **(more than 1)** | 175 (62.9) | 41 (58.6) | 0.50 | 129 (63.2) | 10 (35.7) | 0.005* |
|  | **COPD** | 40 (14.4) | 2 (2.9) | 0.008* | 23 (11.3) | 1 (3.6) | 0.21 |
|  | **Asthma** | 51 (18.3) | 9 (12.9) | 0.28 | 35 (17.2) | 4 (14.3) | 0.70 |
|  | **Diabetes Mellitus** | 80 (28.8) | 17 (24.3) | 0.45 | 45 (22.1) | 7 (25.0) | 0.73 |
|  | **Hypertension** | 131 (47.1) | 31 (44.3) | 0.67 | 105 (51.5) | 9 (32.1) | 0.06* |
|  | **CAD** | 26 (9.4) | 9 (12.9) | 0.38 | 20 (9.8) | 0 (0) | 0.08* |
|  | **CHF** | 24 (8.6) | 5 (7.1) | 0.69 | 21 (10.3) | 2 (7.1) | 0.60 |
|  | **CVA** | 22 (7.6) | 1 (1.4) | 0.094* | 14 (6.9) | 0 (0) | 0.15 |
|  | **Hepatitis B** | 0 (0) | 0 (0) | NA | 0 (0) | 0 (0) | NA |
|  | **Hepatitis C** | 4 (1.4) | 1 (1.4) | 0.99 | 6 (2.9) | 0 (0) | 0.36 |
|  | **Cirrhosis** | 8 (2.9) | 0 (0) | 0.15 | 6 (2.9) | 0 (0) | 0.36 |
|  | **Cancer** | 42 (15.1) | 7 (10.0) | 0.27 | 24 (11.8) | 1 (3.6) | 0.19 |
|  | **Current Chemotherapy** | 7 (2.5) | 2 (2.9) | 0.87 | 2 (1) | 0 (0) | 0.60 |
|  | **CKD** | 35 (12.6) | 10 (14.3) | 0.71 | 28 (13.7) | 3 (10.7) | 0.66 |
|  | **ESRD** | 18 (6.5) | 3 (4.3) | 0.49 | 11 (5.4) | 0 (0) | 0.21 |
|  | **History of solid organ transplant** | 9 (3.2) | 1 (1.4) | 0.42 | 8 (3.9) | 0 (0) | 0.28 |
|  | **Immunodeficiency** | 2 (0.7) | 0 (0) | 0.48 | 3 (1.5) | 0 (0) | 0.52 |
|  | **HIV infection** | 1 (0.4) | 1 (0.3) | 0.62 | 3 (1.5) | 0 (0) | 0.52 |
|  | **Rheumatologic diseases** | 13 (4.7) | 1 (1.4) | 0.22 | 3 (1.5) | 0 (0) | 0.52 |
|  | **Dementia** | 6 (2.2) | 0 (0) | 0.22 | 2 (1) | 3 (10.7) | 0.0009* |
|  | **Peptic ulcer disease** | 0 (0) | 0 (0) | NA | 1 (0.5) | 0 (0) | 0.71 |
|  | **Gastroparesis** | 4 (1.4) | 0 (0) | 0.31 | 0 (0) | 0 (0) | NA |
|  | **Sickle cell disease** | 0 (0) | 1 (1.4) | 0.20 | 0 (0) | 0 (0 | NA |
|  | **Migraine** | 5 (2.2) | 12 (3.4) | 0.37 | 5 (2.5) | 0 (0) | 0.40 |
|  | **Fibromyalgia** | 3 (1.1) | 0 (0) | 0.38 | 1 (0.5) | 0 (0) | 0.71 |
|  | **Chronic pain syndrome** | 10 (3.6) | 4 (5.7) | 0.42 | 10 (4.9) | 0 (0) | 0.23 |
|  | **Alcohol abuse** | 1 (0.4) | 0 (0) | 0.62 | 4 (2.0) | 0 (0) | 0.46 |
|  | **Substance abuse** | 8 (2.9) | 3 (4.3) | 0.55 | 13 (6.4) | 0 (0) | 0.17 |
|  | **Depression** | 32 (11.5) | 0 (0) | 0.003* | 22 (10.8) | 1 (3.6) | 0.23 |
|  | **Psychiatric disease** | 28 (10.1) | 3 (4.3) | 0.13 | 19 (9.3) | 2 (7.1) | 0.71 |
|  | **Pregnancy** | 5 (1.8) | 3 (4.3) | 0.22 | 9 (4.4) | 2 (7.1) | 0.52 |

*SD,* standard deviation*; kg,* kilogram*; BMI*, body mass index; *EMS*, emergency medical services; *GCS*, Glasgow Coma Scale; *SBP*, systolic blood pressure; *DBP*, diastolic blood pressure; *SpO_2_*, oxygen saturation; *AMS*, altered mental status; *SOB*, shortness of breath; *COPD*, chronic obstructive pulmonary Disease; *CAD*, coronary artery disease; *CHF*, congestive heart failure; *CVA*, cerebrovascular accident; *CKD*, chronic kidney disease; *ESRD*, end stage renal disease; *HIV*, human immunodeficiency virus.

Note: * *P*<0.1
